# Spontaneous locomotor activity and L-DOPA-induced dyskinesia are not linked in 6-OHDA parkinsonian rats

**DOI:** 10.3389/fnbeh.2014.00331

**Published:** 2014-10-02

**Authors:** Stefania Sgroi, Alain Kaelin-Lang, Christine Capper-Loup

**Affiliations:** ^1^Department of Neurology and Department of Clinical Research, Movement Disorders Center, Inselspital, Bern University Hospital, and University of BernBern, Switzerland; ^2^Graduate School for Cellular and Biomedical Sciences, University of BernBern, Switzerland; ^3^Neurocentre of Southern SwitzerlandLugano, Switzerland

**Keywords:** Parkinson’s disease, L-DOPA, motor disorders, spontaneous locomotor activity, rotation, dyskinesia

## Abstract

Bradykinesia (slowness of movement) and other characteristic motor manifestations of Parkinson’s disease (PD) are alleviated by treatment with L-dihydroxyphenylalanine (L-DOPA). Long-term L-DOPA treatment, however, is associated with complications such as motor fluctuations and dyskinesia that severely impair the quality of life. It is unclear whether the effect of L-DOPA on spontaneous motor activity and its dyskinesia-inducing effect share a common mechanism. To investigate the possible connection between these two effects, we analyzed the spontaneous locomotor activity of parkinsonian rats before surgery (unilateral injection of 6-OHDA in the right medial forebrain bundle), before treatment with L-DOPA, during L-DOPA treatment (the “ON” phase), and after the end of L-DOPA treatment (the “OFF” phase). We correlated the severity of dyskinesia (AIM scores) with locomotor responses in the ON/OFF phases of chronic L-DOPA treatment at two different doses. We treated three groups of parkinsonian animals with chronic injections of 8 mg/kg L-DOPA, 6 mg/kg L-DOPA, and saline solution and one group of non-lesioned animals with 8 mg/kg L-DOPA. At the end of the experiment, tyrosine hydroxylase (TH) immunoreactivity was analyzed in the striatum of all parkinsonian rats. We found no correlation between the severity of dyskinesia and spontaneous locomotor activity in the ON or OFF phase of L-DOPA treatment. The only observed correlation was between the pathological rotation induced by L-DOPA at the highest dose and locomotor activity in the ON phase of L-DOPA treatment. In addition, a L-DOPA withdrawal effect was observed, with worse motor performance in the OFF phase than before the start of L-DOPA treatment. These findings suggest that different neural mechanisms underlie the effect of L-DOPA on spontaneous motor activity and its dyskinesia-inducing effect, with a different dose-response relationship for each of these two effects.

## Introduction

In Parkinson’s disease (PD), loss of dopaminergic neurons in the pars compacta of the substantia nigra (SN) results in a functional impairment of basal ganglia motor circuits, which leads, in turn, to a characteristic parkinsonian syndrome (Crossman, 1989; Jenner, 2008) including bradykinesia, resting tremor, rigidity, and impairment of posture and gait (Jankovic, 2008; Massano and Bhatia, 2012). L-dihydroxyphenylalanine (L-DOPA) is the most effective drug for exogenous dopaminergic substitution and can alleviate most of the manifestations of PD. Long-term L-DOPA therapy can, however, have disabling complications, including severe fluctuations of motor function (ON/OFF phenomena) and abnormal involuntary movements (AIMs; L-DOPA induced dyskinesia) (Granérus, 1978; Fabbrini et al., 2007). In PD patients, the improvement or worsening of motor symptoms is measured with Part III of the UPDRS (Unified Parkinson’s Disease Rating Scale) in the ON and OFF phases of drug treatment. Typically, the phase without drug intake (OFF) is characterized by manifestations such as delayed gait initiation and bradykinesia (Miyasaki et al., 2002); the phase of drug intake (ON), in which the L-DOPA level is highest, is characterized by the best clinical benefit. Dyskinesia usually arises in the ON phase (Guridi et al., 2012) of chronic L-DOPA treatment and is not seen in the OFF phase. The risk that a patient will develop dyskinesia depends on multiple factors including the degree of dopaminergic neurodegeneration (Obeso et al., 2000), the dose and duration of L-DOPA therapy (Hauser et al., 2006), the age of onset of PD (Kostic et al., 1991; Schrag, 2006), the presence or absence of resting tremor (Kipfer et al., 2011), and genetic factors such as genomic polymorphisms (Linazasoro, 2005). The occurrence of dyskinesia in the phase that is, in all other aspects, the patient’s best motor phase, can severely impair the quality of life (Dodel et al., 2001). In rodent models of PD, many different tests have been proposed for the evaluation of motor impairment (Hamers et al., 2001; Tillerson et al., 2001; Lundblad et al., 2002) after bilateral or unilateral degeneration of dopamine (DA) cells in the SN (Cenci et al., 2002), while the severity of dyskinesia is typically assessed with an analysis of AIMs (Winkler et al., 2002; Cenci and Lundblad, 2007). In many studies, the efficacy of the unilateral lesion of the dopaminergic pathway with 6-hydroxydopamine (6-OHDA) is checked either by spontaneous ipsilateral turning of the animal to the side of lesion or by the number of ipsilateral and contralateral rotations induced by amphetamine or apomorphine injection, respectively (Cenci et al., 1998; Cenci and Lundblad, 2007). Rotation is also a typical ON motor manifestation arising during L-DOPA treatment. Although the causes of drug-induced turning behavior have been investigated in multiple studies (see, e.g., reviews Carta et al., 2006a; Marin et al., 2006), it remains unclear whether rotation should be considered as a type of dyskinesia or as an indication of unilateral hypersensitivity to the 6-OHDA lesion. Behavioral tests such as the cylinder test, the Rotarod (Dekundy et al., 2007), and the CatWalk test (Westin et al., 2012) are widely used to score motor deficits in parkinsonian animals or to quantify motor improvements after L-DOPA treatment. These tests, however, do not reflect a natural spontaneous behavior of animals during the motor performance. In contrast, analysis of the locomotor activity assessed by the open field test (OPF), is a useful method which accounts of the spontaneous exploratory behavior of animals and allows to define a precise time course of the motor activity, for example in the ON and OFF phases of L-DOPA treatment. In general, it is unclear how the effect of L-DOPA on the spontaneous motor activity is related to its effect on dyskinesia. A better understanding of the underlying pathophysiological mechanisms may lead to a better treatment with L-DOPA in order to avoid L-DOPA induced dyskinesia. In this study, we tested the hypothesis of a relationship between dyskinesia and the spontaneous locomotor activity of parkinsonian animals at several phases of L-DOPA treatment and with two different doses of L-DOPA. First, we applied the OPF to assess accurately the motor disturbances that arise before, during, and after L-DOPA therapy. Then, we studied the correlation between the locomotor responses and dyskinesia to determine whether motor activity in the ON and OFF phases is correlated with the severity of dyskinetic movements.

## Materials and methods

### Animals

Twenty eight adult female Sprague-Dawley rats, weighing 210–260 g at the beginning of experiment, were randomly housed in pairs (two rats per cage) from different randomly assigned groups, under a 24-h cycle with 12 h of light and 12 h of darkness with free access to food and water. The room temperature, humidity, and air exchange were automatically controlled. The animals were divided into four groups of seven animals each: three of them (the PD groups) received a unilateral DA-denervating lesion by stereotactic injection of 6-OHDA toxin into the right medial forebrain bundle, as described below; the fourth group (the naive group) did not receive any surgical treatment. Only one rat from the PD groups did not display unilateral dopaminergic lesion and was excluded from data analysis. According to our previous studies, we did not include a sham-surgery group as control group, because we have previously demonstrated that the surgery did not have significant effect on the locomotor performance and on the nigrostriatal dopaminergic system in the sham group (Capper-Loup et al., 2013). All animal procedures were approved by the Animal Research Committee and the Veterinary Office of the Canton of Bern, Switzerland.

### Surgery

The rats were anesthetized intraperitoneally with a mixture of ketamine (75 mg/kg) and xylazine (10 mg/kg). 6-hydroxydopamine (10 μg in 4 μl ascorbic acid 0.1%, Capper-Loup et al., 2005) was injected into the right medial forebrain bundle at the following coordinates in relation to the bregma: posterior 4.0 mm, lateral 1.3 mm, and 7.8 mm ventral to the dura mater, as described by Paxinos and Watson (1986). The 6-OHDA injection was performed at flow rate of 0.5 μl/min. In order to verify the efficacy of the unilateral dopaminergic lesion, all rats were killed at the end of the experiment and the brains processed for tyrosine hydroxylase (TH) immunohistochemistry (see below).

### Experimental design and treatment

The overall PD group (21 rats) was randomly divided into three subgroups. The PD + L-DOPA 8 mg group and the PD + L-DOPA 6 mg group (seven rats each) were given intraperitoneal injections of 8 mg and 6 mg, respectively, of L-DOPA (Sigma-Aldrich, Buchs, Switzerland) together with 15 mg/kg of benserazide (Sigma-Aldrich, Buchs, Switzerland) diluted in NaCl 0.9%, once a day for 21 days, while the PD + saline group (seven rats) received saline injections only. The naive group (seven healthy rats) received the same treatment as the PD + L-DOPA 8 mg group. The experimenter analyzing the behavior was blinded to the injection conditions.

### Spontaneous locomotor test

**Figure 1:**
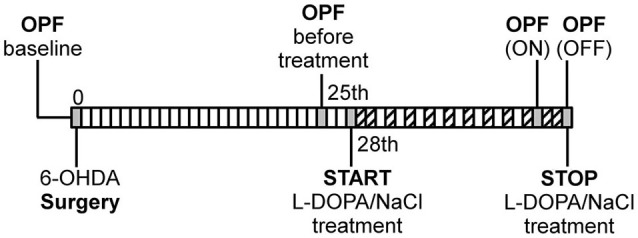
**Schematic illustration of experimental design**. OPF: open field test, (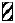: AIM scoring). The first OPF test was performed before surgery as individual baseline measure for each rat.

The spontaneous locomotor activity of all animals was tested in the OPF test during four specific phases (Figure 1): (1) at baseline before surgical lesioning (“before surgery”); (2) 3 days before the start of drug treatment (“before treatment”); (3) at day 19 during L-DOPA or saline injection (the “ON phase”); and (4) at day 22, 1 day after the last drug injection (the “OFF phase”). A pilot study (data not shown) demonstrated that locomotor activity was stable already 2 weeks after lesion in PD rats and we decided to start the behavioral analysis 4 weeks after the surgery to avoid unnecessary stress during the recovery phase of the lesioned animals. To evaluate the relationship between dyskinesia and locomotor activity in the ON phase, we subjected the animals to the OPF test 15 min after the L-DOPA injection, so that the motor variables could be measured while dyskinesia was developing. Two animals were put in adjacent cages and filmed with a video camera. Standard lighting was maintained during the test. The rats performed the OPF test for two periods of 15 min each, separated by a 15 min interval. Videos were analyzed with a computerized tracking system (Ethovision XT 8.5, Noldus, Wageningen, NL) that followed the center of the rat’s body. The automatically analyzed locomotor variables were: “total distance moved”, i.e., the total distance covered in cm by the animals during the whole 15 min observation period; “mean velocity”, the mean of all the velocities calculated for each interval of 0.2 s (time resolution of the system) during the 15 min observation period; the “maximum distance moved”, as the maximal distance traveled in cm by the rats during a single interval of 0.2 s of the 15 min observation period; and “frequency of rotation”, expressed as the number of full (360°) turns taken in the ipsilateral and contralateral directions relative to the lesion (i.e., clockwise and counterclockwise, as the lesion was always made on the right side). We follow the nomenclature of Kalueff et al. (2007) in referring to the total distance moved, mean velocity, and maximum distance moved as variables reflecting horizontal motor activity, and to the frequency of rotation as a variable reflecting turning motor activity.

### Rating of abnormal involuntary movement

For quantification of L-DOPA-induced AIMs (see Figure 1), rats were placed individually in transparent plastic cages and observed by experimenter during the first minute of every 20-min period in the 2 h following the injection of L-DOPA or saline. In addition, a video was taken during the AIMs observation and later analyzed by an investigator blinded to the online rating previously given and to the treatment. Both methods revealed similar results. As described previously (Cenci et al., 1998; Winkler et al., 2002; Cenci and Lundblad, 2007), dyskinesia was classified as one of four subtypes, depending on its bodily distribution: (1) axial AIMs, i.e., lateral flexion of neck and dystonic twisting of trunk contralaterally, i.e., away from the side of lesion; (2) forelimb AIMs, i.e., jerky or dystonic movements of the contralateral forelimb and/or purposeless grabbing movements of the contralateral paw; (3) orolingual AIMs, i.e., purposeless jaw movements and contralateral tongue protrusion; and (4) locomotive AIMs, i.e., increased rotation with contralateral side bias and with at least three paws touching the ground. Each AIM subtype was rated on a severity scale from 0 to 4 (0 = absent, 1 = occasional, i.e., present during less than half of the observation time, 2 = frequent, i.e., present during more than half of the observation time, 3 = nearly continuous with interruption only by strong or external sensory stimuli, 4 = continuous and severe). Aside from this scale, amplitude scores from 0 to 4 were given for each monitoring period (Cenci et al., 1998; Winkler et al., 2002; Carta et al., 2006b). The amplitude of axial AIMs was scored according to the torsional angle of the animal’s neck and upper trunk with the longitudinal axis of the body; that of forelimb AIMs, according to the extent of limb displacement and the visible involvement of proximal muscle groups; that of orolingual AIMs, according to the extent of involvement of facial, masticatory, and tongue muscles and the frequency of jaw opening. All animals were rated on each of 2 days: for each day, a total AIM score was calculated as the sum of the basic score multiplied by the amplitude score for each AIM subtype, excluding the locomotive subtype (Lo) (Carta et al., 2006b; Lundblad et al., 2009). For each day of AIMs observation, the sum of total AIM scores of each subtype previously rated, was used to compare the severity of dyskinesia in the PD + L-DOPA 8 mg and PD + L-DOPA 6 mg groups across the 21 days of L-DOPA treatment (see in the Section Results).

### Tissue preparation and immunohistochemistry

One day after the last injection of L-DOPA/saline treatment and after performing the OPF test in the OFF phase, all rats were intraperitoneally injected with pentobarbital and transcardially perfused with NaCl 0.9% mixed with heparin, followed by 2% paraformaldehyde in phosphate-buffered saline at pH 7.4. The brains were removed, post-fixed for 2 h in the same fixative solution at room temperature and put overnight in 10% sucrose at 4°C. Then the brains were maintained for 3 days in 20% sucrose at 4°C, frozen in isopentane solution and finally stored at −70°C. Coronal sections of 12-μm thickness were cut on a cryostat and stored at −70°C. The brain sections of the PD rats were processed for TH-immunohistochemistry. Briefly, the sections were preincubated for 1 h in blocking serum (5% normal horse serum/0.2% Triton X-100 in phosphate-buffered saline) and were then incubated overnight at 4°C with mouse anti-TH diluted in blocking serum (1:400; Chemicon, Millipore, Temecula, CA, USA). After washing, the sections were incubated for 1 h at room temperature in conjugated goat anti-mouse antibody IRDye800 (1:500; Rockland, Gilbertsville, PA, USA). After the final wash, the sections were allowed to dry before infrared fluorescence scanning (Odyssey, Li-COR, Lincoln, NE, USA).

### Measures of striatal TH density

For the quantification of striatal TH expression, infrared scan was analyzed using the public domain Image J program^1^. The expression of TH labeling in the caudate-putamen region of the ipsilateral DA-denervated side was used to check the accuracy of lesion in two brain sections for each animal, at the rostral level of the striatum (approximately 2.16 mm anterior from the Bregma), according to Paxinos and Watson (1986). The mean gray values were measured for each outlined area and were expressed as the percentage of the averaged values in the contralateral intact side of the PD + saline group set to 100%. Mean values were calculated for each animal and used for the statistical analysis.

### Statistical analysis

To analyze the locomotor variables, we used the mean of the values obtained during the two 15 min observation periods for each animal, expressed as a percentage of the value for that animal that was obtained before surgery (i.e., baseline scale set to 100%). As some of the data were not normally distributed (Shapiro-Wilk test) and did not show homogeneity of variance (Levene test), the statistical analysis was performed with non-parametric tests exclusively. Friedman’s χ^2^ test was performed to evaluate a general effect of the distinct treatment phases (before, during (ON) and after (OFF) drug injection) within groups. Then, the *post hoc* Wilcoxon signed-rank test (Z) was carried out for pairwise comparisons. The Kruskall-Wallis test (H) was performed to assess the general effect of treatment phases across groups. The *post hoc* Mann-Whitney test (U) was performed for groupwise comparisons. The AIM scores were normally distributed with homogeneous variance, and we therefore used ANOVA for repeated measures (repeated factor: time in days) to assess the effect of “time” on “treatment” (two levels: L-DOPA 8 mg, L-DOPA 6 mg) and their interaction. A *post hoc* paired *T*-test was used to evaluate the difference between the treatment days within each group given 8 mg/kg and 6 mg/kg of L-DOPA. Finally, the correlation between the measured motor variables and the AIM scores was analyzed with Spearman’s non-parametric correlation test.

For the analysis of the striatal TH expression, one-way ANOVA was performed to assess the effect of lesion in the DA-denervated side across groups. Then paired *T*-test was used to evaluate the difference between denervated and intact side within each group (significance level was corrected for multiple comparisons). All data were analyzed for a total number of 27 animals, after exclusion of one rat from the PD + saline group because of failure of dopaminergic lesion with 6-OHDA toxin as demonstrated by TH immunohistochemistry (see Section Results). The data are given as mean ± SEM. All analyses were performed with SPSS software (version 15.0, SPSS Inc., Chicago IL, USA). Level of significance was set to *p* < 0.05.

## Results

### Horizontal motor variables

#### Total distance moved and mean velocity

**Figure 2:**
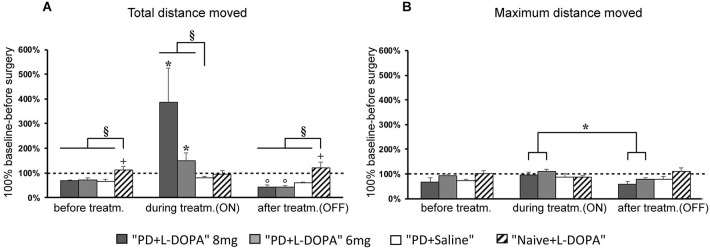
**Horizontal motor activity. (A,B)** Mean of percentage values (± S.E.M.) of the total distance moved and maximum distance moved in the several treatment phases referring to individual values (set to 100%) recorded before surgery. **(A)** The PD + L-DOPA 8 mg and PD + L-DOPA 6 mg groups had more intense locomotor activity in the ON phase than in the other treatment phases (*p < 0.05 vs. before treatment and OFF phase). Both PD + L-DOPA groups had a less intense motor activity in the OFF phase than in the phase before drug treatment (°*p* < 0.05 vs. before treatment). The naive group had a higher distance traveled in the phase before treatment and in OFF phase (^+^*p* < 0.05 vs. the ON phase). Differences between subjects were also seen within each treatment phase (before treatment: §*p* < 0.05 vs. the naive group; ON phase: §*p* < 0.05 vs. PD + saline group; OFF phase: §*p* < 0.05 vs. naive group). **(B)** The PD + L-DOPA 8 mg and PD + L-DOPA 6 mg groups had higher values of maximum distance traveled in the ON phase than the OFF phase (*p < 0.05 vs. the OFF phase).

The analysis of total distance moved and mean velocity revealed different motor responses of the groups across treatment phases. The results obtained for both variables were comparable without any significant difference between them (Figure 2A shows only the data for the total distance moved). All animals lesioned with the 6-OHDA toxin (the overall PD group) had reduced locomotor activity for total distance traveled (PD + L-DOPA 8 mg: 68.2% ± 4.3%; PD + L-DOPA 6 mg: 72.4% ± 8.7%; PD + saline: 66.2% ± 9.0%) and mean velocity (PD + L-DOPA 8 mg: 67.94% ± 4.3%; PD + L-DOPA 6 mg: 72.3% ± 8.7%; PD + saline: 66.1% ± 9.0%) in the “before treatment” phase compared to the “before surgery” phase (with the baseline value set to 100%, as mentioned above). The observed reduction in total distance moved and in mean velocity in each subgroup of the overall PD group differed significantly from that seen in the naive group, as expected (PD + L-DOPA 8 mg: *U* = 1.000, *P* < 0.01; PD + L-DOPA 6 mg: *U* = 6.000, *P* < 0.05; PD + saline: *U* = 4.000, *P* < 0.05). For each of these two motor variables, the observed values varied significantly across treatment phases in the PD + L-DOPA 8 mg group (χ^2^(2) = 12.286, *P* < 0.01) and in the PD + L-DOPA 6 mg group (χ^2^(2) = 14, *P* < 0.001). Both groups had more locomotor activity in the ON phase than before treatment and OFF phase (PD + L-DOPA 8 mg: *Z* = −2.366, *P* < 0.05; PD + L-DOPA 6: *Z* = −2.366, *P* < 0.05 vs. both phases), as well as significantly less movement in the OFF phase compared to before treatment (PD + L-DOPA 8 mg: *Z* = −2.197, *P* < 0.05; PD + L-DOPA 6 mg: *Z* = −2.366, *P* < 0.05). The increase of the motor response in the ON phase was significantly different in the PD + L-DOPA 8 mg and 6 mg groups compared to the PD + saline group (*U* = 0.000, *P* < 0.01; *U* = 5.000, *P* < 0.05, respectively). In the OFF phase, the reduction of motor activity in the PD groups still differed significantly from that in the naive group (PD + L-DOPA 8 mg: *U* = 0.000, *P* < 0.01; PD + L-DOPA 6 mg: *U* = 0.000, *P* < 0.01; PD + saline: *U* = 0.000, *P* < 0.01). Aside from this quantitative motor analysis, we also qualitatively observed that the animals tended to become excited, sniff, and eat immediately after each L-DOPA injection, in conjunction with locomotor hyperactivity. In the OFF phase, in contrast, they tended to rest in one corner of the cage without showing any other interest in conjunction with their reduced locomotor activity.

The motor responses in the PD + saline group did not differ significantly across treatment phases although a small decrease of total distance moved and mean velocity was seen in the OFF phase (total distance moved: 59.74% ± 2.69 (Figure 2A); mean velocity: 59.73% ± 2.67%) compared to the ON phase (total distance moved 79.5% ± 5.8%; mean velocity 79.6% ± 5.8%). All values still remained below the baseline level before surgery (100%). The naive group showed a slight but significant decrease of locomotor activity in the ON phase compared to the before treatment and OFF phases (*Z* = −2.366, *P* < 0.05; *Z*= −2.197, *P* < 0.05, respectively).

#### Maximum distance moved

The experimental groups differed less clearly with respect to the maximum distance moved variable than in the other two variables discussed above (Figure 2B). A significant difference of maximum distance moved across treatment phases was found in the PD + L-DOPA 8 mg group (χ^2^(2) = 8, *P* < 0.05) and the PD + L-DOPA 6 mg group (χ^2^(2) = 10.571, *P* < 0.01). These two groups had higher values in the ON than in the OFF phase (*Z* = −2.366, *P* < 0.05 for both groups; Figure 2B). There was no significant difference of maximum distance moved across treatment phases in the PD + saline group or in the naive group.

### Turning motor variable

#### Ipsilateral and contralateral frequency of rotation

**Figure 3:**
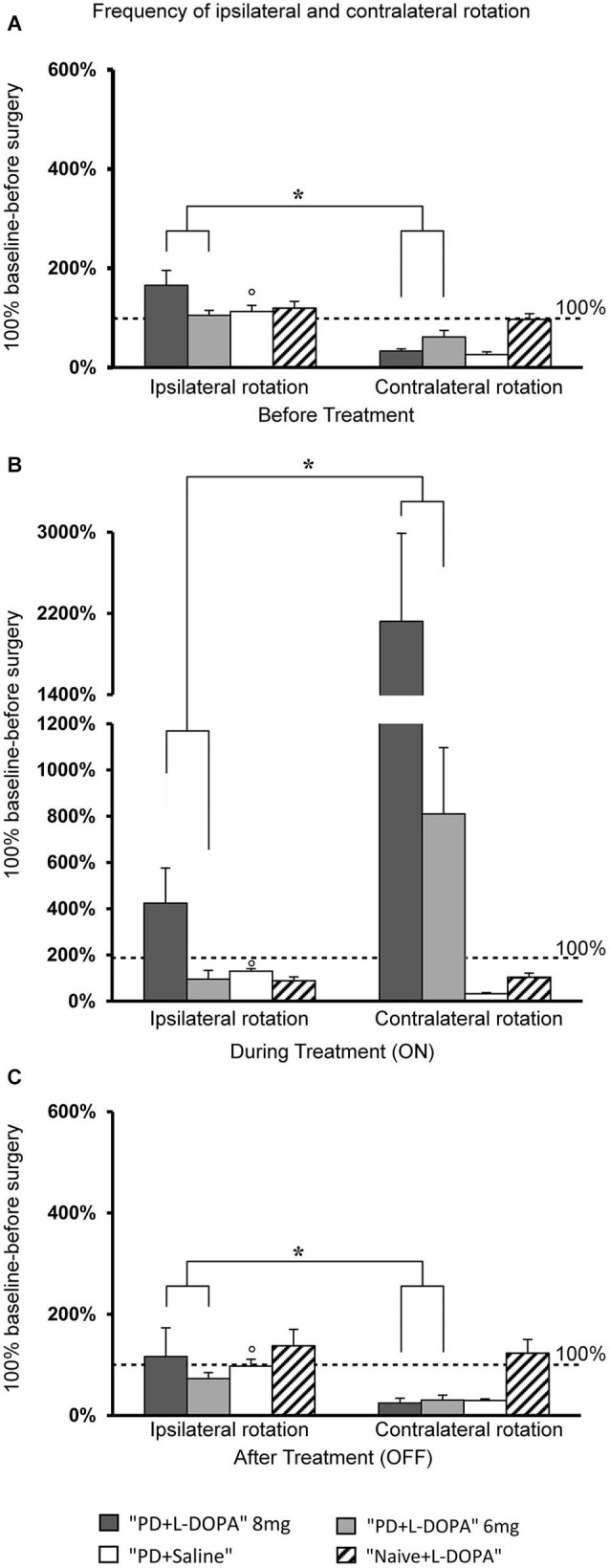
**Turning motor activity. (A–C)** Mean of values (±S.E.M.) of the frequency of rotation in the different treatment phases, expressed as percentages of the individual baseline values (set to 100%) that were recorded before surgery. **(A,C)** The PD + L-DOPA groups showed more frequent ipsilateral rotation before drug treatment and in the OFF phase (*p < 0.05 vs. the contralateral rotation). **(B)** More frequent contralateral rotation was seen in the ON phase (*p < 0.05 vs. ipsilateral rotation). **(A–C)** In contrast, the PD + saline group manifested ipsilateral rotation in all three treatment phases (°*p* < 0.05 vs. contralateral rotations).

To assess turning behavior, the number of full turns (360°) toward and away from the side of the lesion was automatically counted. The frequency of rotation varied across treatment phases in all experimental groups (Figures 3A,B,C). For contralateral rotation, there was a significant difference across treatment phases in the PD + L-DOPA 8 mg group (χ^2^(2)= 11.185, *P* < 0.01) and the PD + L-DOPA 6 mg group (χ^2^(2)= 12.286, *P* < 0.01). Both groups had a higher frequency of ipsilateral rotation in the before treatment and OFF phases (PD + L-DOPA 8 mg: *Z* = −2.366, *P* < 0.05; PD + L-DOPA 6 mg: *Z* = −2.201, *P* < 0.05 vs. contralateral rotation, Figures 3A,C); while contralateral turns were more frequent in the ON phase (PD + L-DOPA 8 mg: *Z* = −2.366, *P* < 0.05; PD + L-DOPA 6 mg: *Z* = −2.366, *P* < 0.05 vs. ipsilateral rotation, Figure 3B). In the PD + saline group, ipsilateral turns were more common in all treatment phases (*Z* = −2.201, *P* < 0.05 vs. contralateral rotation, Figures 3A,B,C).

### Comparison of AIM score in PD groups

**Figure 4:**
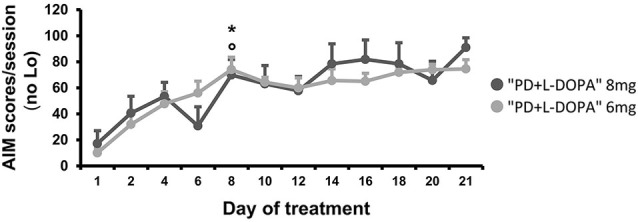
**AIM scores**. Comparison of the bodily (axial, limbic and orolingual) AIM scores developing during chronic treatment at two different L-DOPA dosages. For each day, a total AIM score was calculated as the sum of the basic score multiplied by the amplitude score for each AIM subtype, excluding the locomotive subtype (Lo). The graph expresses the mean of the total AIM scores (±S.E.M.) for all animals during each day of treatment. The severity of the bodily AIMs increased after 1 week (from the eighth day) of daily L-DOPA injections in both treated groups (PD + L-DOPA 8 mg: *p < 0.01 and PD + L-DOPA 6 mg: °*p* < 0.001 vs. the first and second day).

The AIMs occurred only in the PD groups that received daily L-DOPA injections (8 mg and 6 mg), while no dyskinesia was seen in the naive group or in the PD group that was treated only with saline. The severity of AIMs was significantly different across treatment days (effect of time: *F*_(4.805, 57.662)_ = 8.463, *p* < 0.001; Figure 4). Bodily AIMs (axial, limbic, and orolingual subtypes) were more severe from the eighth day of L-DOPA treatment in both of the PD + L-DOPA groups (PD + L-DOPA 8 mg: *t*_(6)_ = −4.384; *p* < 0.01 vs. the first day and *t*_(6)_ = −4.158; *p* < 0.01 vs. the second day; PD + L-DOPA 6 mg: *t*_(6)_ = −7.298; *p* < 0.001 vs. the first day and *t*_(6)_ = −3.825; *p* < 0.01 vs. the second day; Figure 4). After 1 week of daily L-DOPA injections (from the eighth day) the severity of dyskinetic movements reached a plateau value that was maintained similar until the end of treatment days (*p* > 0.05 vs. the 21st day in both PD + L-DOPA groups). Finally, the PD + L-DOPA 8 mg group and the PD + L-DOPA 6 mg group had comparable AIM scores between them and no significant effect time * treatment was found.

### Correlation between motor variables and AIM scores

**Figure 5:**
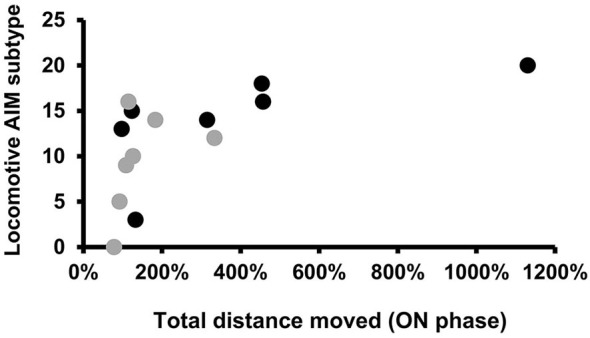
**Spearman’s correlation**. Scatter plots for locomotive AIM subtype, rated on the18th day of L-DOPA treatment, plotted against the total distance moved (expressed in % values) during the ON phase. The graphs represent individual data points for all animals of the PD groups receiving L-DOPA treatment at doses of 8 mg and 6 mg. A significant positive correlation was found only in the PD + L-DOPA 8 mg group. (Spearman’s coefficient:* r* = 0.786, *p* < 0.05).

As it was difficult to rate AIMs accurately during the OPF test, we decided to use for correlation analysis the AIMs rated 1 day before the OPF test in a similar ON phase (on the 18th day of L-DOPA treatment, see Figure 1). No correlation was found between the bodily AIM scores and the locomotor variables recorded in ON and OFF phase of L-DOPA treatment (data not shown). However, there was a significant positive correlation between the Lo and the total distance moved (*r* = 0.786, *p* < 0.05; Figure 5), only in the PD + L-DOPA 8 mg group during the ON phase.

### TH-immunoreactivity

**Figure 6:**
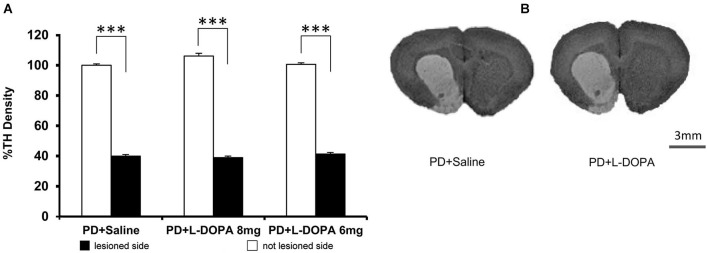
**TH density. (A)** The graph represents the percentage values of TH density (±S.E.M.) in both hemispheres referring to the averaged values in the intact contralateral side of PD + saline group (set at 100%). All PD rats had a significant reduction of the TH density in the DA-denervated side compared to the contralateral side within each group (*** *p* < 0.001 vs. contralateral side). **(B)** Representative image of DA denervation in the right side of the striatum. Scale bar = 3 mm.

As expected, all PD rats showed a severe and significant reduction of TH expression in the 6-OHDA injected side compared to the intact side, confirming the accuracy of the DA lesion (PD + L-DOPA 8 mg: *t*_(6)_ = 45.288 *p* < 0.001; PD + L-DOPA 6 mg: *t*_(6)_ = 60.612 *p* < 0.001; PD + saline *t*_(5)_ = 30.902 *p* < 0.001; Figures 6A,B). Only one rat of the PD + saline group did not present loss of TH fibers in the lesion side and was excluded from data analysis. All PD groups showed a similar loss of TH immunoreactivity without any significant differences across groups.

## Discussion

Our findings demonstrate that there is no relationship between the severity of dyskinesia and the spontaneous locomotor activity in parkinsonian animals during the ON and OFF phases of L-DOPA treatment. In particular, the severity of bradykinesia in the OFF phase was not correlated with the severity of dyskinesia. Only the pathological rotation induced by the higher dose of L-DOPA was found to be correlated with a higher motor response in the ON phase of L-DOPA treatment. Our behavioral results suggest that two different neural mechanisms are involved in the development of dyskinesia and the control of motor performance. Although many studies have already investigated the motor changes and the dyskinesia in parkinsonian animal model, for our knowledge this is the first study that assesses in parallel, the spontaneous locomotor responses of parkinsonian animals at specific phases of L-DOPA treatment and correlates these motor responses with the severity of dyskinetic movements.

### Correlation of rotation movements induced by L-DOPA and motor parameters

In parkinsonian rats treated with L-DOPA, the locomotor activity and the movement velocity were higher during the ON phase of L-DOPA treatment than before and after treatment, as expected. We used two low L-DOPA doses (6 and 8 mg/kg respectively) that are comparable to the doses used in humans and that are known to induce AIMs in parkinsonian animals (Lundblad et al., 2002; Winkler et al., 2002). Despite the small difference between the two dosages, they clearly differed in their propensity to induce contralateral rotation with increased locomotor activity in the ON phase. The rats treated with 8 mg of L-DOPA had a more intense motor response and more frequent contralateral turns than those treated with 6 mg, although the degree of DA denervation did not differ across the two PD + L-DOPA groups. Nonetheless, the severity and progression of dyskinesia were similar in the two groups.

To assess whether the increased locomotor behavior was linked with, or perhaps due to, the rotational movements (locomotive AIM scores) or, alternatively, with classic bodily dyskinesia (body AIM), which closely resembles the choreatic dyskinesia seen in humans, we analyzed the relationship between the AIM scores and the locomotor variables of the parkinsonian rats treated with L-DOPA (at higher and lower doses) in the ON and OFF phases. Only the rotational movements measured with the AIM score and induced by L-DOPA at the 8 mg dose were correlated with more intense locomotor activity during the ON phase. Individual variability in the motor responses to higher dose of L-DOPA in the PD + L-DOPA 8 mg group, likely explains the correlation observed only in this group, between the rotational movements and the values for the distance traveled. Our results strongly confirm that slightly different L-DOPA dosages can have different effects on spontaneous motor performances without influencing the severity of dyskinesia. As previously suggested (Lundblad et al., 2002), pathological contralateral rotation should not be regarded as a valid measure of dyskinesia (Lundblad et al., 2002), but rather as a manifestation of the hypersensitivity of the lesioned side to L-DOPA treatment (Ungerstedt, 1976; Tolwani et al., 1999). Our findings are consistent with these previous findings and suggest that the neural mechanisms leading to dyskinesia differ from those that mediate spontaneous motor responses, with different dose-effect relationships. A previous study on rats that were made parkinsonian with 6-OHDA lesioning did not reveal any significant correlation between amphetamine-induced rotation and motor behavior (Metz and Whishaw, 2002). Further studies are needed to improve our understanding of the pathways responsible for the motor and dyskinetic manifestations. Conceivably, the direct and indirect pathways, which are differently modulated by chronic L-DOPA treatment, might be involved in the control of locomotor activity and in the development of the involuntary movements, respectively.

### Motor and non-motor responses in parkinsonian rats after L-DOPA withdrawal

Interestingly, in both groups of parkinsonian rats treated with L-DOPA, locomotor activity was lower after L-DOPA was discontinued (the OFF phase) than before starting L-DOPA treatment. This state of hypomobility resembles the OFF motor state of parkinsonian patients after L-DOPA is stopped and the therapeutic effect of the drug wears off. We hypothesize that the decreased motor performance in the OFF phase, only seen in the two PD groups given L-DOPA, might be due to a combined motor and non-motor effect of L-DOPA withdrawal, and not just to the freezing of gait that often reflects the OFF condition (Jankovic, 2005; Devos et al., 2010). In this study, we qualitatively observed an overall “excited” behavioral state a few minutes after each L-DOPA injection in the ON phase and, in contrast, a quiet behavioral state in the OFF phase. In a possibly related finding, studies of non-motor manifestations in patients with PD have shown that chronic L-DOPA treatment may promote the development, not just of motor fluctuations, but of mood fluctuations as well (Maricle et al., 1995; Kulisevsky et al., 2007). Significant mood changes have been found to be associated with ON/OFF phenomena in patients with PD (Nissenbaum et al., 1987; Menza et al., 1990). Fluctuating euphoria (76%) and hyperactivity (94%) were found in the ON motor state, while anxiety was more common in the OFF motor state (88%) (Witjas et al., 2002). The observation and analysis of spontaneous animal behavior, as in our study, may be a useful tool for the assessment of both motor and non-motor effects of L-DOPA treatment but it cannot differentiate between both. Specific tests for anxiety and for a depressive state would be needed for a better and specific assessment of non-motor manifestations.

### No motor behavioral changes in the naive group

In this study, we also analyzed the effect of L-DOPA in healthy rats. Locomotor activity was similar before and after L-DOPA treatment while in the ON phase, a reduction of the motor state might be due to the side effects of chronic L-DOPA treatment, such as nausea, that frequently also develop in parkinsonian patients. In accordance with observations on healthy subjects (Newman et al., 1985) or subjects with essential tremor erroneously treated with L-DOPA, our findings confirm that therapeutic doses of L-DOPA have no effect on the locomotor performance or the dyskinesia development in healthy rats. This confirms that the extent of striatal dopaminergic denervation is an important determinant of the pharmacological activity of L-DOPA and the development of its secondary effects, including dyskinesia (Cenci and Lundblad, 2006; Putterman et al., 2007).

## Conclusion

This study shows that the severity of dyskinesia is not correlated with the locomotor activity in the ON and OFF phases of L-DOPA treatment; rather, only the rotational movements induced by higher dose of L-DOPA are correlated with higher locomotor activity in the ON phase. In addition, a combined motor and non-motor L-DOPA withdrawal effect could explain a lower motor performance in the OFF phase of L-DOPA treatment compared to the phase before starting the L-DOPA treatment. The analysis of spontaneous animal locomotor activity with the OPF test is a useful methodological approach for analyzing accurately behavioral responses that resemble the motor disturbances of human patients with PD at several phases of L-DOPA treatment.

## Conflict of interest statement

The authors declare that the research was conducted in the absence of any commercial or financial relationships that could be construed as a potential conflict of interest.

## Footnotes

^1^http://imagej.nih.gov/ij

## Abbreviations

6-OHDA: 6-hydroxydopamine
AIMs: abnormal involuntary movements
DA: dopamine
L-DOPA: L-dihydroxyphenylalanine
OPF: open field
PD: Parkinson’s disease
TH: tyrosine hydroxylase.
